# Music Improves Subjective Feelings Leading to Cardiac Autonomic Nervous Modulation: A Pilot Study

**DOI:** 10.3389/fnins.2017.00108

**Published:** 2017-03-10

**Authors:** Satoshi Kume, Yukako Nishimura, Kei Mizuno, Nae Sakimoto, Hiroshi Hori, Yasuhisa Tamura, Masanori Yamato, Rika Mitsuhashi, Keigo Akiba, Jun-ichi Koizumi, Yasuyoshi Watanabe, Yosky Kataoka

**Affiliations:** ^1^Cellular Function Imaging Team, Division of Bio-function Dynamics Imaging, RIKEN Center for Life Science TechnologiesKobe, Japan; ^2^Department of Physiology, Osaka City University Graduate School of MedicineOsaka, Japan; ^3^Health Metrics Development Team, Integrated Research Group, RIKEN Compass to Healthy Life Research Complex Program, RIKEN Cluster for Science and Technology HubKobe, Japan; ^4^Center for Health Science Innovation, Osaka City UniversityOsaka, Japan; ^5^Pathophysiological and Health Science Team, RIKEN Center for Life Science TechnologiesKobe, Japan; ^6^Health Evaluation Team, RIKEN Compass to Healthy Life Research Complex Program, RIKEN Cluster for Science and Technology HubKobe, Japan; ^7^Department of Medical Science on Fatigue, Osaka City University Graduate School of MedicineOsaka, Japan; ^8^Della Inc.Tokyo, Japan; ^9^Biosystem Engineering, Faculty of Engineering, Yokohama National UniversityYokohama, Japan

**Keywords:** subjective feelings, fatigue, healing, music, cardiac autonomic function

## Abstract

It is widely accepted that listening to music improves subjective feelings and reduces fatigue sensations, and different kinds of music lead to different activations of these feelings. Recently, cardiac autonomic nervous modulation has been proposed as a useful objective indicator of fatigue. However, scientific considerations of the relation between feelings of fatigue and cardiac autonomic nervous modulation while listening to music are still lacking. In this study, we examined which subjective feelings of fatigue are related to participants' cardiac autonomic nervous function while they listen to music. We used an album of comfortable and relaxing environmental music, with blended sounds from a piano and violin as well as natural sound sources. We performed a crossover trial of environmental music and silent sessions for 20 healthy subjects, 12 females, and 8 males, after their daily work shift. We measured changes in eight types of subjective feelings, including healing, fatigue, sleepiness, relaxation, and refreshment, using the KOKORO scale, a subjective mood measurement system for self-reported feelings. Further, we obtained measures of cardiac autonomic nervous function on the basis of heart rate variability before and after the sessions. During the music session, subjective feelings significantly shifted toward healing and a secure/relaxed feeling and these changes were greater than those in the silent session. Heart rates (ΔHR) in the music session significantly decreased compared with those in the silent session. Other cardiac autonomic parameters such as high-frequency (HF) component and the ratio of low-frequency (LF) and HF components (LF/HF) were similar in the two sessions. In the linear regression analysis of the feelings with ΔHR and changes in LF/HF (ΔLF/HF), increases and decreases in ΔHR were correlated to the feeling axes of Fatigue-Healing and Anxiety/Tension–Security/Relaxation, whereas those in ΔLF/HF were related to the feeling axes of Sleepiness–Wakefulness and Gloomy–Refreshed. This indicated that listening to music improved the participants' feelings of fatigue and decreased their heart rates. However, it did not reduce the cardiac LF/HF, suggesting that cardiac LF/HF might show a delayed response to fatigue. Thus, we demonstrated changes in cardiac autonomic nervous functions based on feelings of fatigue.

## Introduction

Listening to music is generally recognized as a way to regulate people's various feelings and mood/emotional states toward relaxation, sleepiness, motivation, and sadness. Indeed, human feelings are easily transformed by music. Listening to music as therapy reportedly mediates prolonged fatigue and pain reduction (Krout, 2001; Chuang et al., 2010; Graversen and Sommer, 2013; Archer et al., 2015; Mercadíe et al., 2015) and has been widely used to treat people with health and psychiatric disorders worldwide (Lin et al., 2011). Half the general population in modern society experiences fatigue and its sensations caused by continual stress and prolonged deficiency of rest or sleep (Watanabe and Kuratsune, 2006). Fatigue and its sensations are pre-symptoms described as indicating a deterioration of performance and attentiveness in social activities and work (Watanabe, 2008). There are a variety of fatigue states such as chronic, physical, mental, complex (a combination of physical and mental fatigue), and inflammatory fatigue resulting from a variety of daily fatigue loadings, and these states are often inconsistent with perception of the fatigue condition. Recently, cardiac autonomic nervous modulation has been proposed as a characteristic feature of mental, acute, and daily levels of fatigue, based on the correlation of scores on the Chalder Fatigue Scale, a common fatigue index (Mizuno et al., 2011; Tanaka et al., 2011), indicating a specific nature of this relationship between cardiac autonomic nervous modulation and fatigue. Moreover, the modulation has been used to assess prolonged fatigue and pain in clinical studies (Logier et al., 2006; Chuang et al., 2010). To this end, to reduce the accumulation of daily fatigue and its sensations, it is effective to control the cardiac autonomic nervous functions through the use of suitable music. Listening to music is practical as it is low-cost and -risk.

Music expresses various feelings such as healing, relaxation, and arousal, and it prompts heart rate variability (HRV) and autonomic nervous system. Different kinds of music lead to different responses involving these feelings. For example, rock and heavy metal music are often used to create arousal, particularly among the youth. The autonomic nervous system, combined with the sympathetic and parasympathetic nerves, largely acts unconsciously and regulates various bodily functions such as heart rate, digestion, respiratory rate, and pupillary response. In recent times, the concept of autonomic nervous modulation has been applied to healthcare management in various fields other than medical treatment (Lucini et al., 1997; Singh et al., 2004; Sakuragi and Sugiyama, 2006; Mizuno et al., 2011; Tanaka et al., 2011) and to the risk management of diseases such as arrhythmia and cardiovascular disease (Zimmerman et al., 1988; Barutcu et al., 2014; Zafrir et al., 2016). Autonomic nervous activation in a large range of emotions was previously reported. Emotion is a specific and strong feeling, such as anger, anxiety, fear, sadness, and happiness. It causes changes in subjective feeling quality, expressive behavior, and physiological activation (Kreibig, 2010). However, there is still no scientific consensus on the distinct patterns of autonomic nervous activation and/or modulation across physiological outcome variables allowing for the identification of an emotional state. Thus, it still remains unclear which subjective feelings of fatigue, including healing, fatigue, sleepiness, and a refreshed feeling, are quantitatively correlated to cardiac autonomic nervous functions while when a person listening to music.

It has not adequately been shown how subjective feelings of fatigue are affected by environmental music or how these feelings are related to the cardiac autonomic nervous modulations in the body. In this study, we recruited office workers as participants and examined their subjective feelings as affected by environmental music, after their daily work shift ended. We attempted to monitor real-time subtle changes in their subjective feelings and states of mind using the KOKORO scale, a frequently used, self-reporting subjective mood measurement system on smartphones and/or tablet devices. The system can easily record subtle changes in subjective feelings as a person listens to music. We measured cardiac autonomic nervous modulations from HRV, one of the fatigue indexes, before and after the sessions. Next, we investigated the relation between the participants' subjective feelings of fatigue and cardiac autonomic nervous modulations while they listened to environmental music.

## Materials and methods

### Experimental design

In all, 20 healthy participants [34 ± 5.4 years of age (mean ± SDM), 12 females and 8 males] were enrolled. All experiments were performed after the participants' daily work shift (i.e., after 5 p.m.). All the participants underwent both environmental music and silent sessions. The environmental music was provided by Della Inc. (Tokyo, Japan) and is now commercially available. The environmental music album used in the experiment was composed by Mitsuhiro. It consisted of comfortable and relaxing music with blended sounds from a piano, violin, harp, acoustic guitar, and synthesizer. Some of the music included natural sound sources such as breeze, rushing creeks, and birdsongs, which are thought to bring about relief.

**Figure 1:**
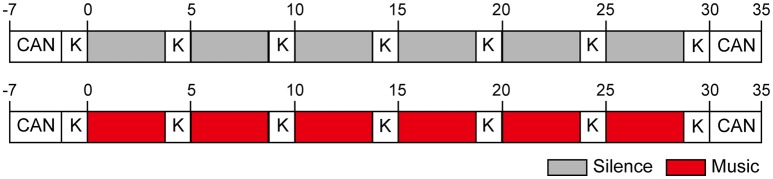
**Experimental periods in the silent and environmental music sessions (30 min each)**. Subjective feelings for each participant were evaluated seven times, including 2 min before the beginning of each session, using the KOKORO scale. For 5 min before and after each session, cardiac autonomic nervous modulations were measured using the Fatigue Measurement Device: from −7 to −2 min and 30 to 35 min, on the basis of a zero point at the beginning of each session. K and CAN indicate the evaluation of subjective feelings and measurements of cardiac autonomic nervous modulations, respectively. The boxes in red and gray indicate periods of listening to the environmental music (Music) and to silence (Silence), respectively.

A crossover trial of environmental music and silent sessions was performed three times for each participant. Each experimental period in either type of session lasted 30 min, and all experiments for each participant were conducted over 6 days. In both the sessions, the participants' subjective feelings were evaluated every 5 min, for a total of seven times, including 2 min at the beginning of each session, using the KOKORO scale (Figure 1). For 5 min before and after each session, cardiac autonomic nervous modulations were measured using a Fatigue Measurement Device: from −7 to −2 and 30 to 35 min, on the basis of a zero point at the beginning of each session (Figure 1). Whether the participants were listening to environmental music or silence, they wore noise-canceling headphones in each experiment. The study was performed in a quiet room. The study protocol was approved by the Ethics Committee of the Center for Health Science Innovation (Osaka City University), and all the participants provided written informed consent. We paid a participation honorarium to each subject. The honorarium was also approved by the Ethics Committee of the Center for Health Science Innovation.

### Measurements of subjective feeling using the KOKORO scale

The KOKORO scale was developed by the RIKEN Center for Molecular Imaging Science (Kobe, Japan) to capture subtle changes in mood and emotional states, such as the “feeling of security,” “anxiety,” and “motivation.” The scale is constructed as a four-quadrant matrix in a two-dimensional space (a panel size of 10 × 10 cm) and can easily record subjective feelings and states of mind through touch input on the screens of smartphones and tablet devices. This application of the devices quantifies subjective feelings without using language, such as would be necessary in written questionnaires.

**Figure 2:**
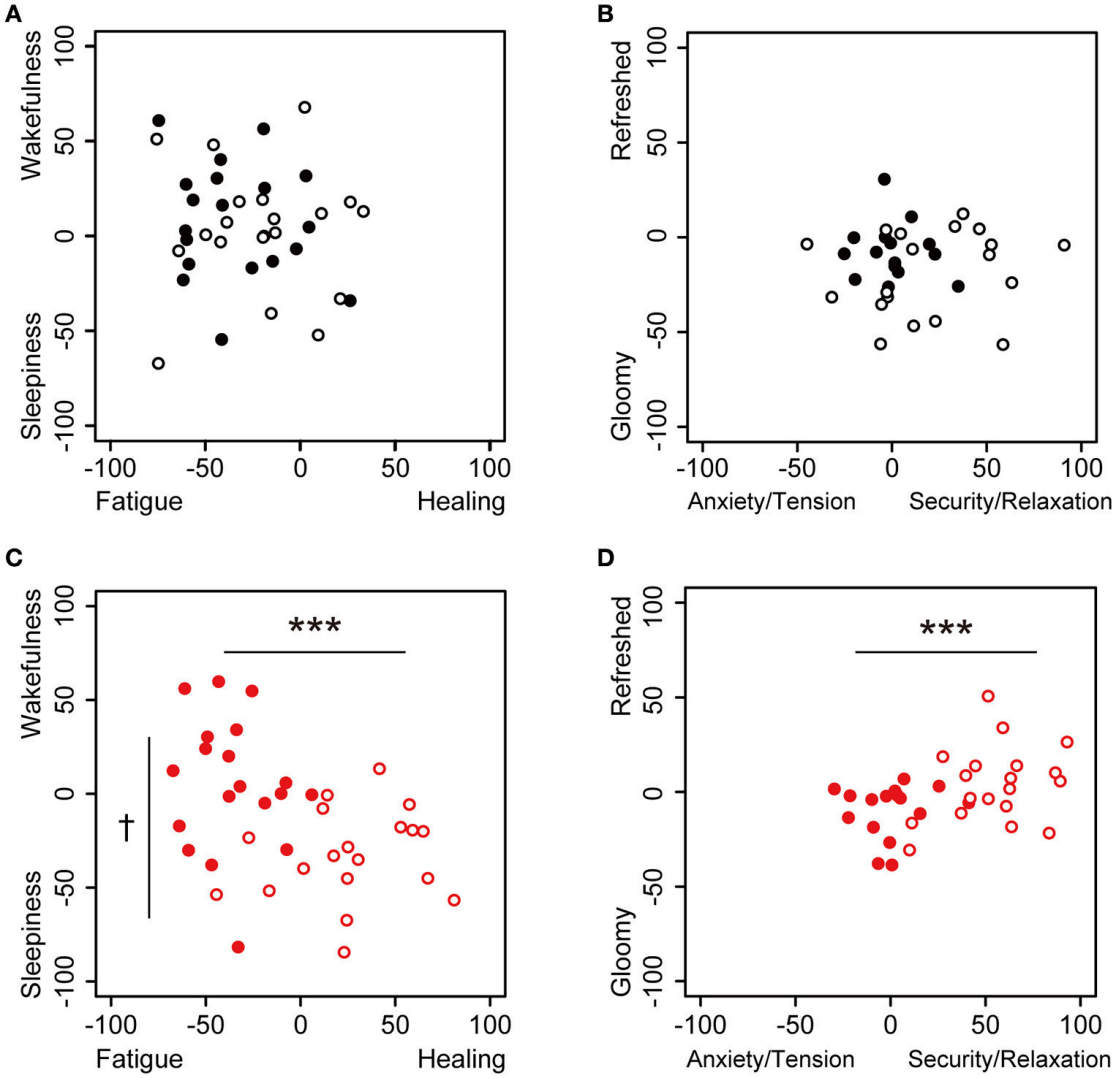
**Dot plot of the values of subjective feelings at 0 and 30 min in the sessions**. Subjective feelings in the environmental music and silent session are plotted. **(A)** The touch panel was set to Fatigue-Healing on the *x*-axis and Sleepiness-Wakefulness feeling on the *y*-axis, and data are presented for the beginning (closed, *n* = 20) and 30 min (open, *n* = 19) in the silent session. **(B)** The panel was set to Anxiety/Tension–Security/Relaxation on the *x*-axis and Gloomy-Refreshed feeling on the *y*-axis, and data are presented for the beginning (closed, *n* = 17) and 30 min (open, *n* = 19) in the silent session. **(C)** The panel was set to Fatigue-Healing on the *x*-axis and Sleepiness–Wakefulness feeling on the *y*-axis, and data are presented for the beginning (closed, *n* = 19) and 30 min (open, *n* = 19) in the music session. **(D)** The panel was set to Anxiety/Tension–Security/Relaxation on the *x*-axis and Gloomy-Refreshed feeling on the *y*-axis, and data are presented for the beginning (closed, *n* = 17) and 30 min (open, *n* = 19) in the music session. Data were analyzed using a two-way ANOVA. ^***^p < 0.01: significantly different than at the zero point at the beginning of the music session on the *x*-axis. ^†^*p* < 0.1: significantly different than at the zero point at the beginning of the music session on the *y*-axis. Data in the silent session did not show any significant differences.

We used the KOKORO scale to measure the participants' subjective feelings. We constructed two types of KOKORO scale panels with the use of standardized scales; one square panel of the KOKORO scale was set to “Fatigue–Healing” feelings on the *x*-axis and “Sleepiness–Wakefulness” feelings on the *y*-axis, and the other panel was set to “Anxiety/Tension–Security/Relaxation” feelings on the x-axis and “Gloomy–Refreshed” feeling on the *y*-axis, as shown in Figure 2. The coordinate data for both axes were quantified as actual numbers from −100 to 100 (continuous values) on the basis of where the participants touched the panel; the centers of the axes were set to zero. The measurements of subjective feeling using the KOKORO scale were performed seven times during each session, as shown in Figure 1.

### Cardiac autonomic nervous function measurements

For testing cardiac autonomic nervous functions, spectral frequency domain analysis for variability of the time interval between R waves (RR-interval) has been performed using electrocardiography (ECG) since the 1980s (Akselrod et al., 1981; Appel et al., 1989). Various indexes of autonomic nervous control, including the basis of an interbeat interval (IBI), heart rates (HR), heart rate variability (HRV), standard deviation of the interbeat intervals (SDNN), root mean square of successive differences (RMSSD), mean of the absolute value of the difference between successive interbeat intervals (MSD), mean square successive difference (MSSD), respiratory sinus arrhythmia (RSA), cardiac sympathetic index (CSI), and cardiac vagal index (CVI) were reported (Allen et al., 2007). In many studies of fatigue, parameters of low-frequency (LF) component, high-frequency (HF) component, and LF/HF were used for the indexes of cardiac autonomic nervous modulation (Tanaka et al., 2009, 2011, 2015; Mizuno et al., 2010, 2011, 2014; Park et al., 2011, 2012; Sommerfeldt et al., 2011; Leti and Bricout, 2013; Yu et al., 2013; Schmitt et al., 2015; Vigo et al., 2015). LF and HF components indicate the cardiac autonomic nervous modulations likely influenced by the parasympathetic nervous system (Houle and Billman, 1999; Reyes del Paso et al., 2013).

In this study, a Fatigue Measurement Device VM302 (Hitachi Systems, Ltd., Tokyo, Japan) was used to evaluate changes in the cardiac autonomic nervous functions of each participant. We used ECG signals and evaluated cardiac autonomic nervous modulations by an indirect measurement of cardiac autonomic nerve activity. When ECG was missing, photoplethysmography was used for the calculations. During the measurement of cardiac autonomic nervous function, the participants remained at rest, and kept their eyes closed.

By monitoring HRV in ECG, we collected the 5 min data of cardiac autonomic nervous function with a 600 Hz sample rate. The built-in firmware in VM302 detected the R wave peaks using an algorithm of peak detection based on a hill-climbing method, and the obtained peak time on R waves was transmitted to an external computer. From each R peak time, a time series of R-R intervals was sequentially generated. Noise of digital signals was removed using a low pass filter, whereas a removal of linear trends for R-R intervals was not performed in the measurements.

We extracted 30 s of R-R interval sequences from the 5 min collected data in every heart beat event after 30 s: Total number of extracted sequences were “total number of heart beats in 5 min” minus “the heart beat number in the beginning 30 s.” The collected data were analyzed using MemCalc/Win (GMS Co., Ltd., Tokyo, Japan). The heart rate (HR) was calculated from the inverse of R-R intervals in each heart beat. Frequency analyses for R-R interval variation were performed with the maximum entropy method (MEM), which is capable of estimating the power spectrum density from short time series data and is adequate for examining changes in HRV in different conditions of short duration (Takusagawa et al., 1999; Kanaya et al., 2003). In addition, instead of a stationary test of R-R intervals fluctuation, outliers in the 30 s data of R-R intervals were defined as different values by 0.75-fold lower and 1.75-fold higher of the median values of R-R intervals for 30 s, and the matched data of R-R intervals was removed.

The power spectral density obtained after MEM was divided into LF component, within the range of 0.04–0.15 Hz, and HF component, within the range of 0.15–0.4 Hz. The mean values of HR, LF, HF, and LF/HF obtained in each time series were calculated as representative values in each measurement. Changes in cardiac autonomic nervous modulations, such as ΔHR, ΔLF, ΔHF, and ΔLF/HF, were calculated as differences between before and after the sessions for each participant.

### Data analysis

We designed three-time crossover experiments (totaling six experiments) for each participant. Representative values for each participant in the sessions were obtained by the mean of the three experiments. Outliers in the obtained dataset were defined as the points that were different from lower and upper quartiles by 1.5-fold lower and higher values of the interquartile range, respectively, and the outlier data were removed from further analysis.

Statistical analyses were performed using the *R* statistics platform (R Foundation for Statistical Computing, http://www.r-project.org; R Core Team, 2016). Statistically significant differences between the subjective feelings data were evaluated using a two-way (time and session) analysis of variance (ANOVA) and *post-hoc* Tukey's honest significant difference test. Effect sizes were also calculated after the ANOVA. Significant differences for cardiac autonomic data were calculated using the paired Welch's test.

A simple linear regression analysis between cardiac autonomic nervous modulations and subjective feelings was performed using the *R* statistics platform. Pearson's correlation coefficient (*r*) was calculated between the parameters of ΔHR and ΔLF/HF and the changes in subjective feelings. Values of *p* were obtained by a test of no correlation.

## Results

### Changes in subjective feeling by listening to environmental music

We used comfortable and relaxing environmental music, with blended sounds and natural sound sources. Changes in the participants' subjective feelings were measured in the environmental music and silent sessions using the application of the KOKORO scale (Figure 1). The subjective feelings of “Fatigue,” “Healing,” “Sleepiness,” “Wakefulness,” “Anxiety/Tension,” “Security/Relaxation,” “Gloomy Feeling,” and “Refreshed Feeling” at 0 and 30 min for the two sessions are shown in Figure 2; this panel is similar to the panel in the KOKORO scale. In the silent sessions, subjective feelings were almost identical at 0 and 30 min (Figures 2A,B), whereas in the music sessions, they were significantly altered in the direction of healing, sleepiness, security/relaxation, and refreshed feeling (Figures 2C,D). The negative values of the Fatigue–Healing axis at 0 min indicated that several subjects felt fatigue, which we assume is due to the timing of the experiment at the end of the workday.

**Figure 3:**
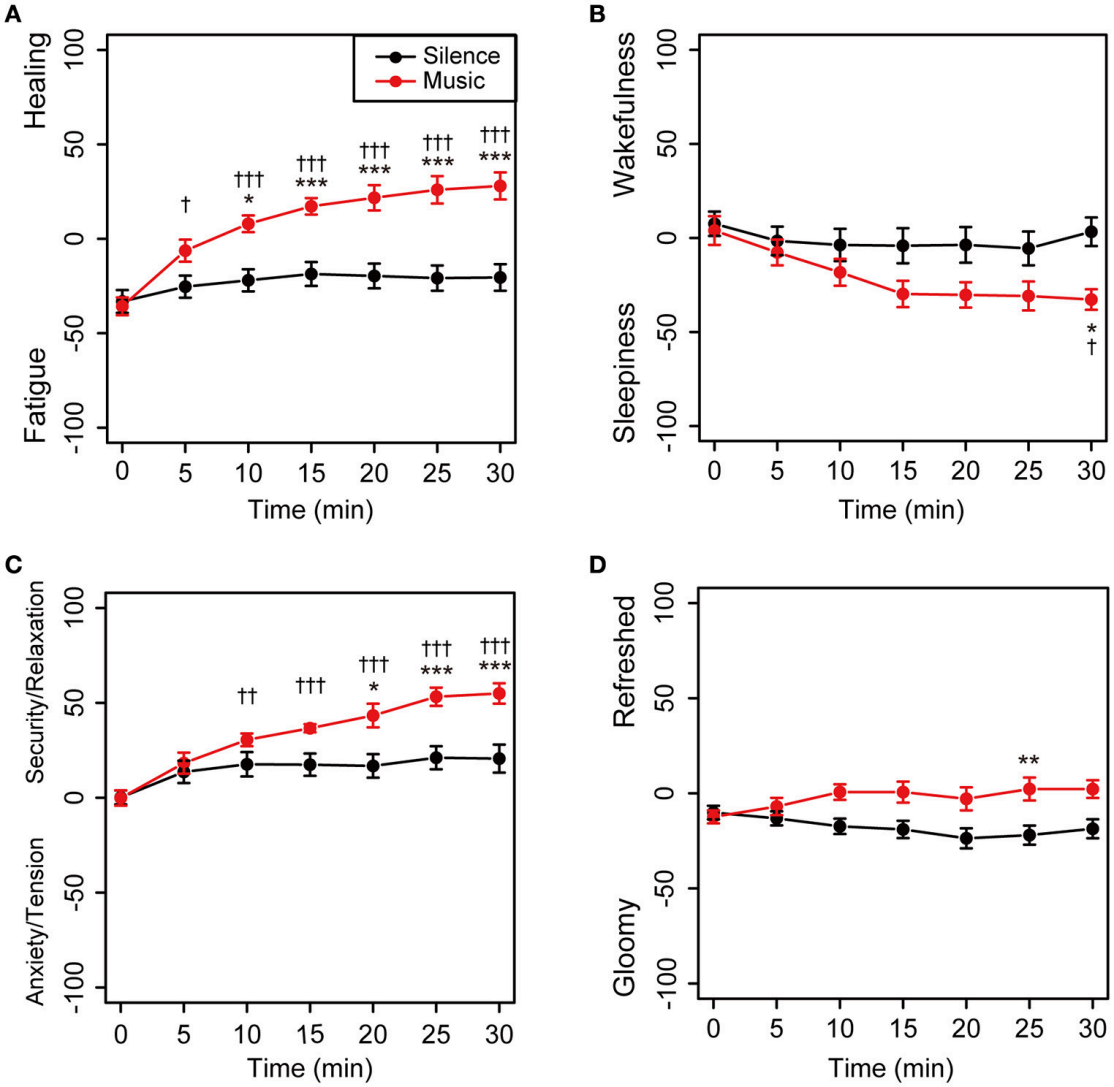
**Time-dependent changes in subjective feelings for all participants**. Subjective feelings were measured using the KOKORO scale seven times during each session for the 20 participants. The results of the Fatigue–Healing axis **(A)**, Sleepiness–Wakefulness feeling axis **(B)**, Anxiety/Tension–Security/Relaxation axis **(C)**, and Gloomy–Refreshed feeling axis **(D)** are shown. Coordinate data are recorded as actual consecutive numbers from –100 to 100. Black and red closed circles are shown for the silent and music sessions, respectively. Data are represented as mean ± SEM and analyzed using a two-way ANOVA. ^***^p < 0.01, ^**^p < 0.05, and ^*^p < 0.1: significantly different between the silent session and the music session at the same time. ^†††^*p* < 0.01, ^††^*p* < 0.05, and ^†^*p* < 0.1: trend significantly different than at the zero point at the beginning of the music session. Data in the silent session did not show any significant differences.

The time-dependent changes in subjective feelings in both the sessions are shown in Figure 3. Descriptive statistics of the self-reported subjective feelings in both sessions was summarized in Supplementary Table 1. In the environmental music session, subjective feelings in the Fatigue–Healing and Anxiety/Tension–Security/Relaxation axes were gradually shifted toward the direction of more healing and greater security/relaxation, with statistical significance (Figures 3A,C). The feelings in the silent session did not show shifts (Figure 3). In the Sleepiness–Wakefulness feeling axis, the feelings were significantly shifted toward sleepiness 15 min into the music session and then remained unchanged until the 30 min mark (Figure 3B). The mean values of the Sleepiness–Wakefulness axis in the silent session were almost constant from the beginning of the measurement. The feelings on the Gloomy–Refreshed feeling axis showed little change in both the sessions (Figure 3D).

In addition, as the participants were thrice exposed to a repeated measurement design, an experimental setting (music session) and control setting (silent session), we examined the relevance of the measurement design. Changes in subjective feelings during the silent and music sessions showed a similar trend in the three times of measurements (Supplementary Figure 1 in Data Sheet 1), and the effect size of each session was almost consistent (Supplementary Table 2). These results indicated that environmental music significantly influences feelings such as healing, sleepiness, and security/relaxation.

**Figure 4:**
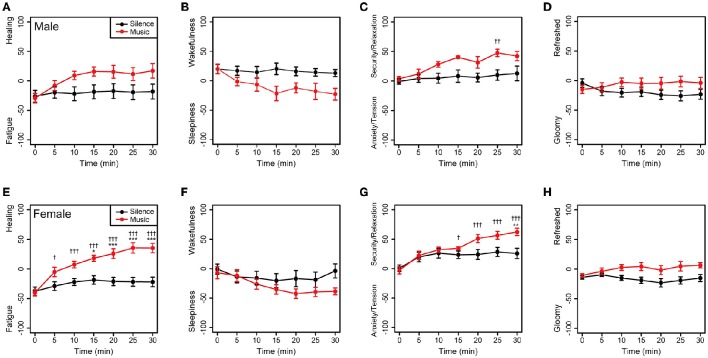
**Changes in subjective feelings of the male and female participants during the experimental period**. **(A–D)** Changes in the subjective feelings of the male group (*n* = 8) in each session. **(E–H)** Changes in the subjective feelings of the female participants (*n* = 12) in each session. Black and red closed circles are shown for the silent and music sessions, respectively. Data are represented as mean ± SEM and analyzed using a two-way ANOVA. ^***^p < 0.01, ^**^p < 0.05, and ^*^p < 0.1: significantly different between the silent session and the music session at the same time. ^†††^*p* < 0.01, ^††^*p* < 0.05, and ^†^*p* < 0.1: trend significantly different than at the zero point at the beginning of the music session. Data in the silent session did not show any significant differences.

To investigate gender difference, the subjective feelings of the male and female participants were examined. As shown in Figure 4, changes in the subjective feeling of each group showed a similar tendency in comparison to the results of all participants. This showed there was little gender difference in feelings in both the music and silent sessions.

### Cardiac autonomic nervous modulations caused by listening to environmental music

**Figure 5:**
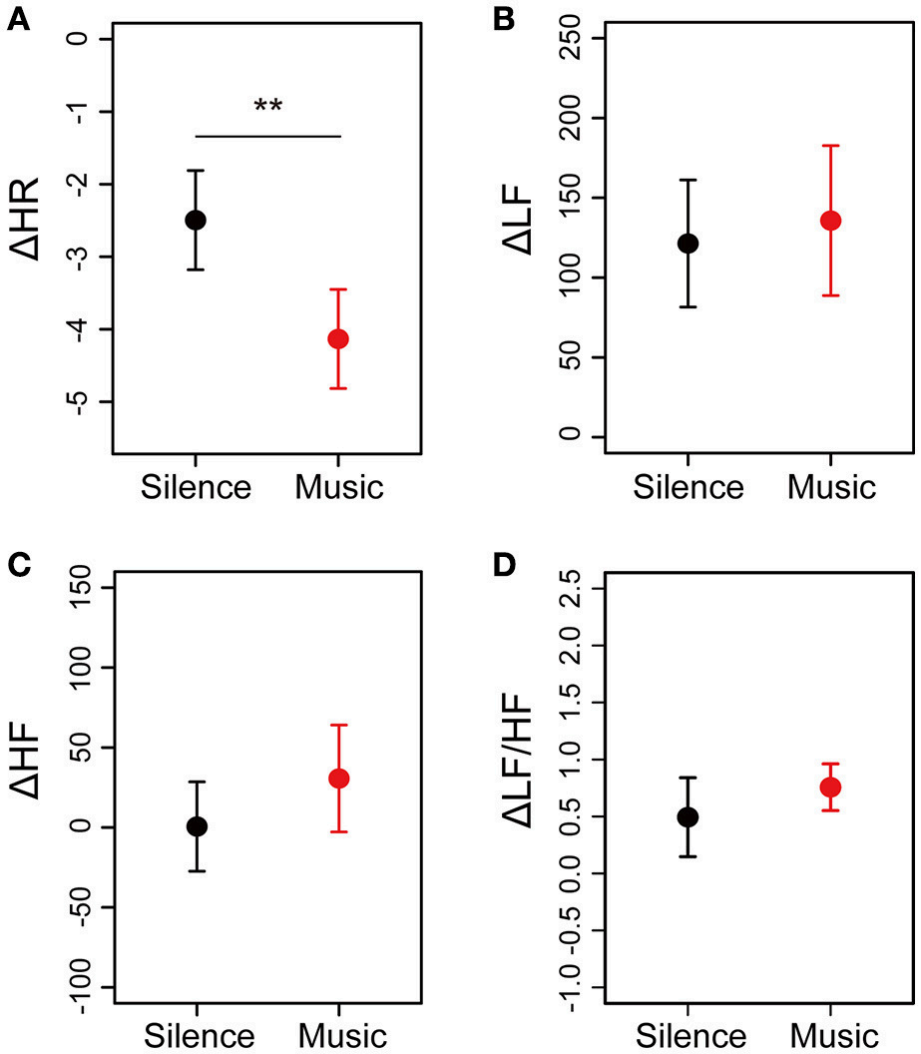
**Differences in cardiac autonomic nervous modulations between the silent and music sessions for all participants**. Cardiac autonomic measurements of the 20 participants were performed twice, before, and after the sessions. Differences in HR, LF, HF, and LF/HF between before and after the session, which are defined as ΔHR, ΔLF, ΔHF, and ΔLF/HF, respectively, were calculated. Averaged values of ΔHR **(A)**, ΔLF **(B)**, ΔHF **(C)**, and ΔLF/HF **(D)** for each participant are plotted. Data are represented as mean ± SEM. ^**^p < 0.05: trend significant difference between the silent session and the music session.

To evaluate the cardiac autonomic nervous modulations caused by listening to the environmental music, cardiac autonomic measurements were performed before and after the sessions (Figure 1). After calculating the averaged differences in HR (ΔHR), LF power (ΔLF), HF power (ΔHF), and the LF/HF ratio (ΔLF/HF) between before and after sessions, the differences in cardiac autonomic parameters were plotted in Figure 5. Descriptive statistics of the cardiac autonomic parameters in both sessions was summarized in Supplementary Table 3. ΔHR-values in the music session showed significant decreases compared with those in the silent session (Figure 5A). Cardiac autonomic parameters, such as ΔLF, ΔHF, and ΔLF/HF, did not show a difference between either of the sessions (Figures 5B–D). Moreover, we identified a gender difference in the cardiac autonomic nervous modulations (Supplementary Figure 2 in Data Sheet 1). In the male and female groups, ΔHR showed decreases between the silent and music sessions (Supplementary Figures 2A,E). Averaged differences in ΔLF and ΔHF between the music and silent sessions are almost identical for the two gender groups (Supplementary Figures 2B,C,F,G). ΔLF/HF showed a shift to a different direction in the male and female groups (Supplementary Figures 2D,H). The results suggested little differences of cardiac autonomic nervous modulations in response to the music between the male and female groups.

### Subjective feelings discriminated by patterns of cardiac autonomic nervous modulations caused by listening to environmental music

**Figure 6:**
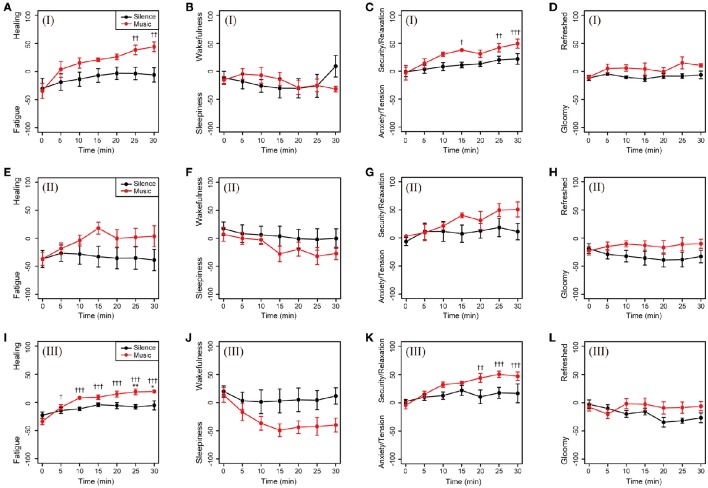
**Subjective feelings discriminated by patterns of cardiac autonomic nervous modulations in the music session**. Participants were classified into four patterns from the plus and/or minus sign of ΔLF/HF and ΔHF. **(A–D)** Class I: decrease in ΔLF/HF and increase in ΔHF (four participants). **(E–H)** Class II: increase in ΔLF/HF and decrease in ΔHF (five participants). **(I–L)** Class III: increase in ΔLF/HF and increase in ΔHF (six participants). Class IV: decrease in ΔLF/HF and decrease in ΔHF (one participant, data not shown). Four participants were assigned as outliers, and their data were removed before the analysis. Black and red closed circles are shown for the silent and music sessions, respectively. Data are represented as mean ± SEM and analyzed using two-way ANOVA. ^**^p < 0.05 and ^*^p < 0.1: significantly different between the silent session and the music session at the same time. ^†††^*p* < 0.01, ^††^*p* < 0.05, and ^†^*p* < 0.1: trend significantly different than at the zero point at the beginning of the music session. Data in the silent session did not show any significant differences.

To investigate the relations between the patterns of cardiac autonomic nervous modulations and subjective feelings, we classified the 20 participants into four patterns according to their increases and/or decreases in ΔLF/HF and ΔHF: class I, decrease in ΔLF/HF and increase in ΔHF (four participants, Figures 6A–D); class II, increase in ΔLF/HF and decrease in ΔHF (five participants, Figures 6E–H); class III, increase in ΔLF/HF and increase in ΔHF (six participants, Figures 6I–L); and class IV, decrease in ΔLF/HF and decrease in ΔHF (one participant, data not shown). Furthermore, four participants were excluded due to the removal of outlier data points. On the Fatigue–Healing axis, the subjective feeling of classes I and III in the music session significantly shifted in the direction toward healing with a larger change compared to those of the other classes (Figures 6A,E,I). On the Sleepiness–Wakefulness axis, the participants in class III became sleepier during the music session (Figures 6B,F,J). On the relaxation axis, the subjective feelings of classes I and III showed a significant shift to relaxation during the music session (Figures 6C,G,K). In the Gloomy–Refreshed feeling axis, participants in class I showed a slight shift to the positive values of refreshed feelings after the music session (Figures 6D,H,L). These results indicated different behaviors of subjective feelings corresponding to patterns of cardiac autonomic nervous modulation.

**Figure 7:**
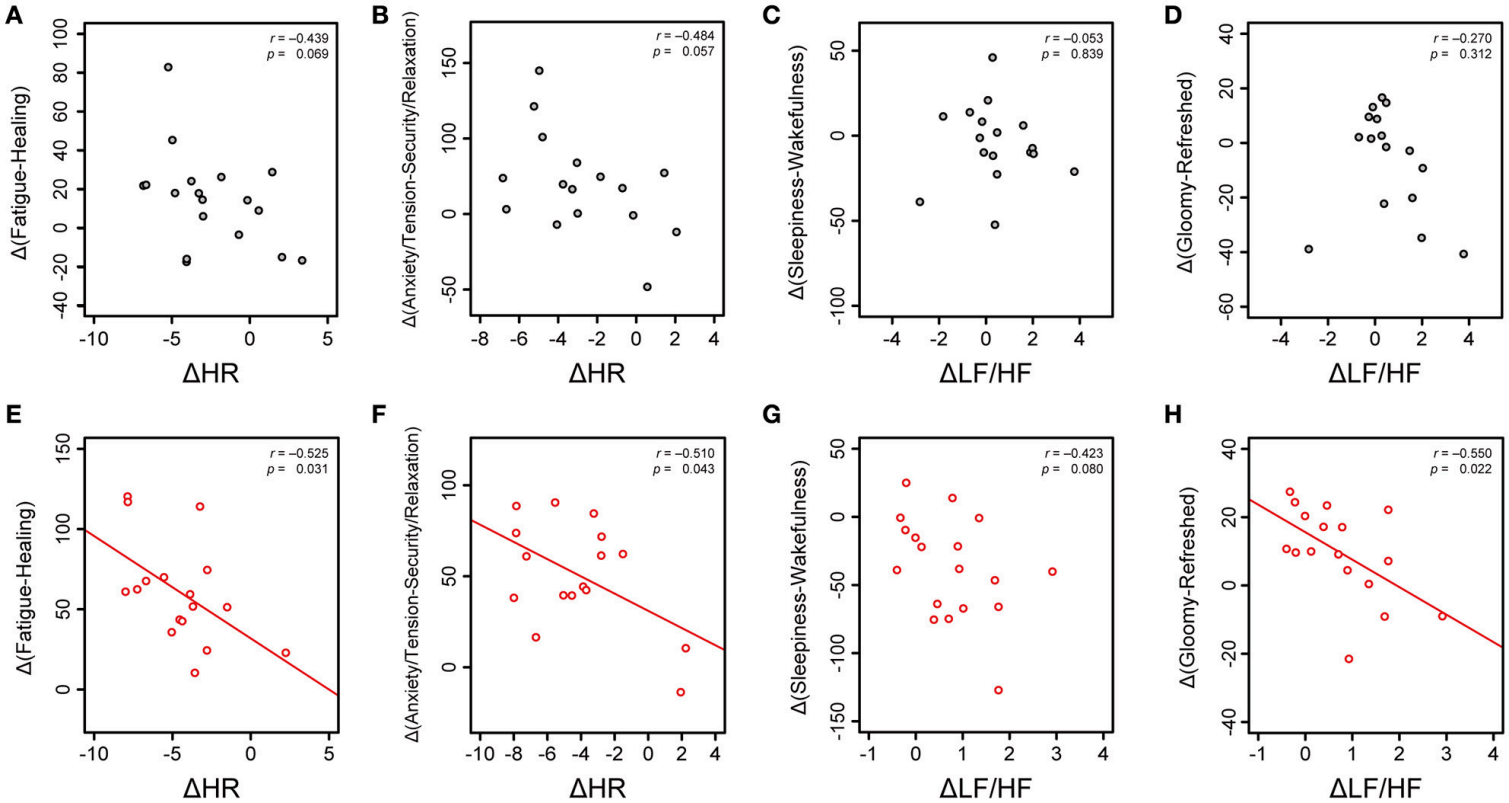
**Relations between cardiac autonomic nervous modulations and subjective feelings**. Changes in subjective feelings are assumed as the differences from the zero point at the beginning of the session to 30 min. In the silent session, changes in HR before and after the session (ΔHR) are plotted against Δ(Fatigue–Healing) at 30 min **(A)** and Δ(Anxiety/Tension–Security/Relaxation) at 30 min **(B)**. Changes in the LF/HF ratio before and after the session (ΔLF/HF) are plotted against Δ(Sleepiness–Wakefulness) at 30 min **(C)** and Δ(Gloomy–Refreshed) at 30 min **(D)**. In the music session, ΔHR-values are plotted against Δ(Fatigue–Healing) at 30 min **(E)** and Δ(Anxiety/Tension–Security/Relaxation) at 30 min **(F)**. ΔLF/HF-values are plotted against Δ(Sleepiness–Wakefulness) at 30 min **(G)** and Δ(Gloomy–Refreshed) at 30 min **(H)**. Linear regression lines (red lines), Pearson's correlation coefficients (*r*), and statistical significances, *p*-values, are shown.

Next, using the variation of cardiac autonomic parameters such as ΔHR and ΔLF/HF between before and after the sessions, we performed linear-regression analysis to predict changes in subjective feelings. The correlation coefficients are listed in Supplementary Table 4. For the silent session, representative plots of changes in subjective feelings against ΔHR and ΔLF/HF are shown in Figures 7A–D and no significant and/or weak correlations in those relations were observed. In the music session, the values of ΔHR were significantly correlated to the Δ(Fatigue-Healing) values (Figure 7E, *r* = −0.525, *p* < 0.031) and Δ(Anxiety/Tension-Security/Relaxation) values (Figure 7F, *r* = −0.510, *p* < 0.043). The values of ΔLF/HF showed a weak correlation with Δ(Sleepiness–Wakefulness) values (Figure 7G, *r* = −0.423, *p* < 0.080) and a significant correlation with Δ(Gloomy–Refreshed) values (Figure 7H, *r* = −0.550, *p* < 0.022). The correlation analysis revealed that the values of ΔHR in the music session were significantly correlated to recovering fatigue feelings and anxiety/tension feelings, whereas increases in ΔLF/HF contributed to increases in gloomy feelings (decreases in refreshed feelings).

## Discussion

In this study, we investigated the relations between subjective feelings and cardiac autonomic nervous modulations during environmental music and silent sessions. Cardiac autonomic nervous functions were examined by spectral frequency domain analysis of RR-intervals in ECG. Changes in HR represent a balance of the sympathetic and parasympathetic nervous systems. In addition to HR, we calculated LF, HF, and LF/HF as other indexes of cardiac autonomic nervous modulations. LF and HF have been discussed to likely relate to the parasympathetic nervous systems (Reyes del Paso et al., 2013) by some experiments: Physiological measurements including blood pressure, respiration, and autonomic nervous discharge indicate that HF component is mainly mediated by parasympathetic nervous modulation (Malliani et al., 1991); pharmacological blockade of the vagal nerve remarkably reduces LF component (Pomeranz et al., 1985); sympathetic blockade has no significant effects on LF component (Taylor et al., 1998); LF component is determined mainly by the parasympathetic nervous system in cardiac regulations (Reyes del Paso et al., 2013).

We demonstrated that subjective feelings from fatigue to healing were improved by the participants' listening to environmental music (Figures 2, 3). The examination of subjective feelings revealed non-significant gender differences in the male and female participants (Figures 4). Further, cardiac autonomic nervous modulations, such as ΔLF, ΔHF, and ΔLF/HF did not show any significant changes between the music and silent sessions (Figures 5B–D), but ΔHR values after the music sessions were significantly decreased (Figure 5A). We found that the changes in subjective feelings of fatigue while listening to music are apparently correlated with those in ΔHR and ΔLF/HF as cardiac physiological responses (Figure 7). These results indicated that listening to the environmental music improved the participants' feelings of fatigue accompanied by sensitive decreases in heart rates, whereas it did not reduce cardiac LF/HF. This difference might show a delayed cardiac autonomic nervous response to fatigue.

Several previous studies have demonstrated that listening to music was effective in improving mood, decreasing anxiety, and increasing self-rated relaxation (Davis and Thaut, 1989). Also, several pleasant types of music and/or silence subjectively induced higher relaxation and comfort, as well as lower fatigue, compared to unpleasant music (Krout, 2001; Hirokawa and Ohira, 2003; Iwanaga et al., 2005; Chuang et al., 2010; Mercadíe et al., 2015). When participants listened to relaxing and comforting music, HR was significantly lower than when they listened to exciting music (Nater et al., 2006). Moreover, the HF component was higher for sedative music than for excitative music, similar to that for silence (Iwanaga et al., 2005). These results are almost consistent with those of the present study. Taken together, the results suggest that listening to environmental music is effective for improving subjective feelings, such as fatigue, and for maintaining mental healing and a refreshed feeling.

Furthermore, we examined which subjective feelings of fatigue, including healing, fatigue, sleepiness, and a refreshed feeling, were quantitatively correlated to cardiac autonomic nervous modulations when the participants listened to music. We found a significant correlation of ΔHR with the feelings of Fatigue–Healing and Anxiety/Tension–Security/Relaxation in the music session but not in the silent session (Figure 7). Also, our results suggested that the variations in cardiac nervous variability, especially patterns of LF/HF and HF, are predictive of changes in subjective feelings. In the classes defined in this study, variations of changes in subjective feelings were observed; decreases in ΔLF/HF contribute to increases in healing and refreshed feelings and decreases in sleepiness feeling (Figure 6). The linear-regression analysis of ΔLF/HF during the music session suggested that the changes from wakefulness to sleepiness and from gloomy to refreshed feeling mainly contributed to cardiac autonomic nervous functions. Indeed, these results might point to the prediction of cardiac autonomic nervous modulations during listening to music through the monitoring and quantifying of various subjective feelings. It was previously reported that in the respective relations between fatigue and cardiac autonomic nervous function, LF and the LF/HF ratio were positively correlated with the daily level of fatigue, as evaluated by the Chalder Fatigue Scale (Tanaka et al., 2011). In addition, HF was negatively correlated with fatigue score. Another study showed that both the visual analog scale score for fatigue sensation and the LF/HF ratio after an 8 h fatigue session inducing mental fatigue significantly increased, compared to before the session, in addition to which the HF component after the fatigue session significantly decreased (Mizuno et al., 2011). In the case of participants with chronic fatigue syndrome, who show substantial fatigue unrelieved by rest, cardiac autonomic parameters were reported to be strong predictors of stress levels and subjective sensations of fatigue (Boneva et al., 2007; Burton et al., 2010). Taken together, the reduction in fatigue sensations as a result of listening to the music is associated with the parasympathetic predominance in the cardiac autonomic nervous system.

On the contrary, our results also suggest the relation of sleepiness to cardiac autonomic nervous functions. Sleepiness and sleep quality are known to relate both to the LF/HF ratio and the HF component; increasing the LF/HF ratio and decreasing HF parameters significantly during sleep lead to low sleep quality (Burton et al., 2010). This suggests the correlation of relevant mental variables with sleep quality and cardiac autonomic nervous dysfunction. Thus, in the music session, feelings of sleepiness together with the imbalance of cardiac autonomic nervous modulations may suppress mental healing and refreshed feelings. The imbalance of cardiac autonomic nervous modulations may be considered to refer to the accumulation of daily stimulation for cardiac autonomic nervous function and unmatched music with each pasted memory or taste. Our data may provide a future challenge to the understanding of the relations between subjective feelings and cardiac autonomic nervous modulations in daily life. Further, we would like to try to evaluate cardiac autonomic nervous modulations using other autonomic indexes such as IBI, SDNN, RMSSD, MSD, MSSD, RSA, CSI, and CVI in future studies of fatigue.

The environmental music used in this study improved subjective feelings related to fatigue and anxiety/tension in almost all participants. The classification of the variability of cardiac autonomic nervous modulations allowed us to obtain the variation in subjective feelings. In the simple linear regression analysis, we found a correlation of subjective feelings with some cardiac autonomic nervous modulations during the participants' time listening to music. This suggested an expectation of changes in cardiac autonomic nervous functions based on the subjective feelings of fatigue. Our study allows us to consider health management through the evaluation of the variability of the cardiac autonomic nervous system and to quantify variation in subjective sensations.

## Author contributions

Conceived and designed the study: YK. Produced music: RM and KA. Experiments: YK and HH. Data analysis: SK and YK. Drew figures and created tables: SK, YN and NS. Contributed to the writing of this manuscript: SK, YN, KM, HH, YT, MY, JK, YW and YK.

## Funding

Funding for this research was received from Della Inc. (Tokyo, Japan). This work was partially supported by the Management Expenses Grant for the RIKEN Center for Life Science Technologies.

### Conflict of interest statement

This study was funded by Della Inc. (Tokyo, Japan). RM and KA were employees of Della Inc., which has commercialized newly developed environmental music. This study was designed by YK, and the sponsor had no control over the interpretation, writing, or publication of this work. The corresponding author had full access to all the data in the study and had final responsibility for the decision to submit for publication. The other authors declares that the research was conducted in the absence of any commercial or financial relationships that could be construed as a potential conflict of interest.
