# Supplementary material for: Music Improves Subjective Feelings Leading to Cardiac Autonomic Nervous Modulation: A Pilot Study
Source: Front Neurosci. 2017 Mar 10;11:108. doi: 10.3389/fnins.2017.00108 (PMC5344927; doi:10.3389/fnins.2017.00108)
Supplement: Supplementary file 5 [file DataSheet1.docx]

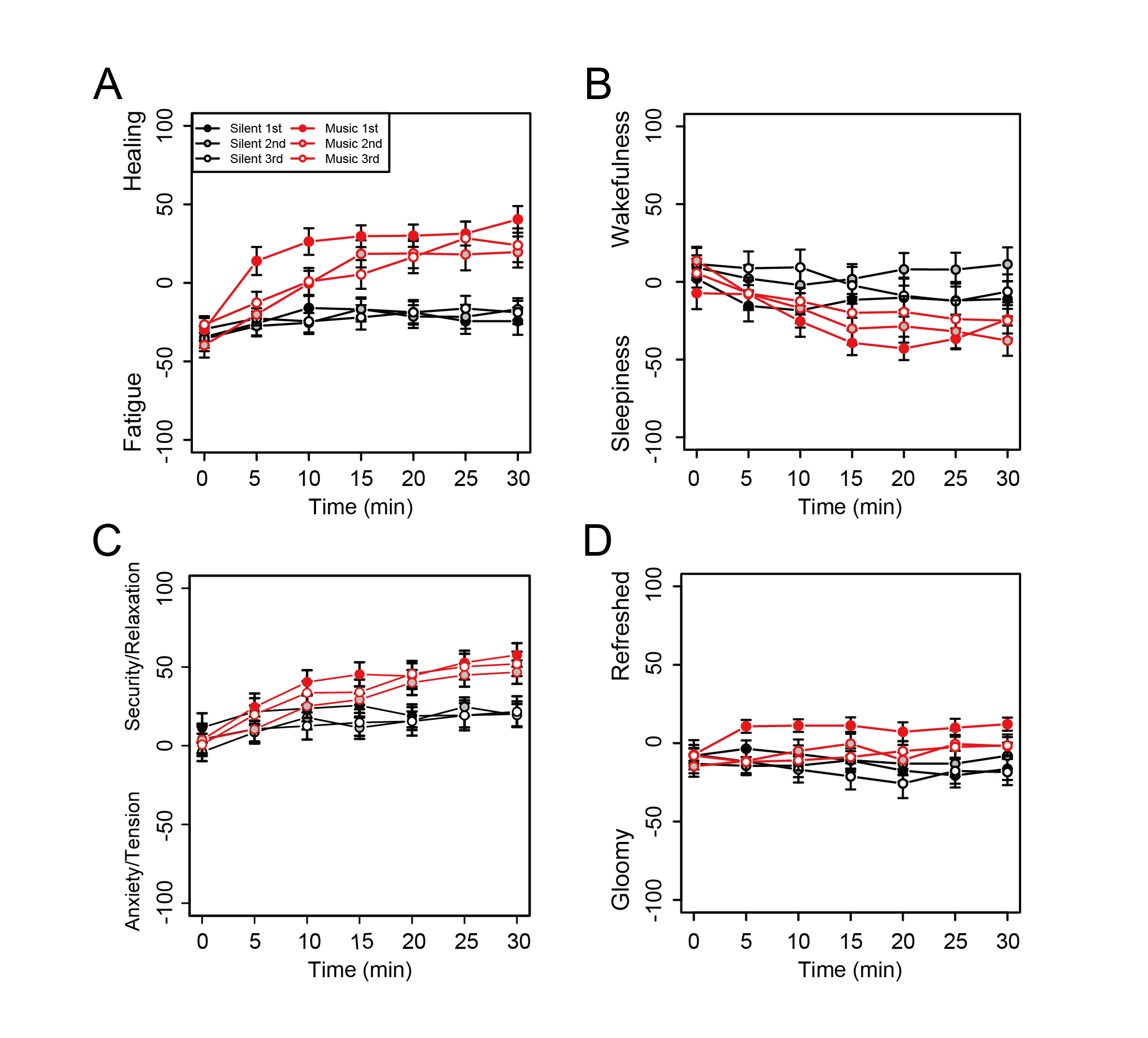


Supplementary Figure 1. Time-dependent changes in subjective feelings in each measurement.

Three times, subjective feelings were measured using the KOKORO scale, at seven points during each session for the 20 participants. Each result of the Fatigue–Healing axis (A), Sleepiness–Wakefulness feeling axis (B), Anxiety/Tension–Security/Relaxation axis (C), and Gloomy–Refreshed feeling axis (D) is shown. The black and red circles represent the silent and music sessions, respectively. The color-closed, gray-closed, and open circles represent the 1^st^, 2^nd^, and 3^rd^ measurement, respectively. The data are represented as mean ± SEM.


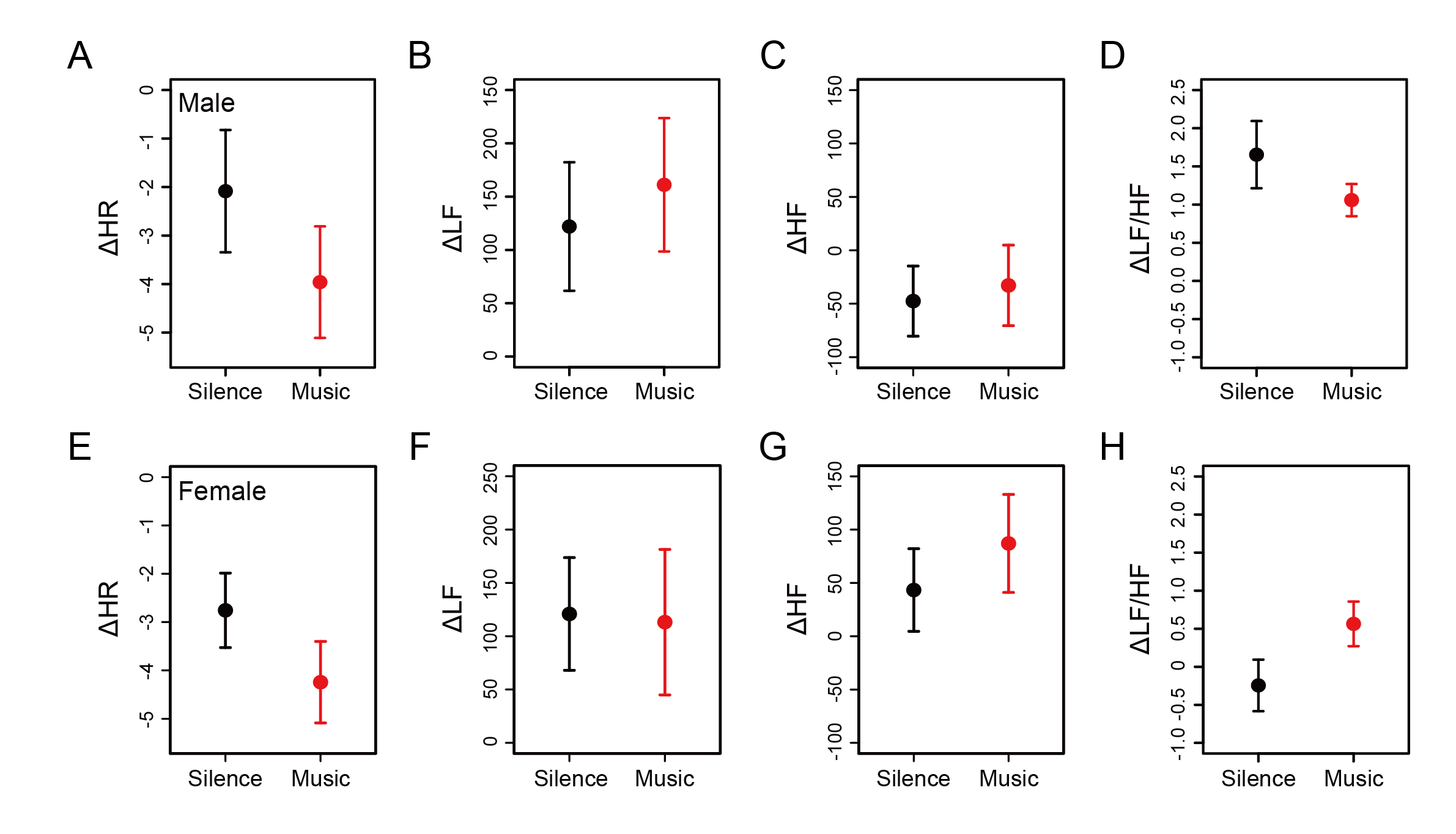


Supplementary Figure 2. Gender differences in the cardiac autonomic modulations in the silent and music sessions.

(A–D) Differences of cardiac autonomic modulations such as ΔHR (A), ΔLF (B), ΔHF (C), and ΔLF/HF (D) in the silent and music sessions for the male participants (n = 8). (E–H) Differences of cardiac autonomic activities such as ΔHR (E), ΔLF (F), ΔHF (G), and ΔLF/HF (H) in the silent and music sessions for the female participants (n = 12). The data are represented as mean ± SEM. The data in both sessions did not show any significant differences.
